# A Nonword Repetition Task Discriminates Typically Developing Italian-German Bilingual Children From Bilingual Children With Developmental Language Disorder: The Role of Language-Specific and Language-Non-specific Nonwords

**DOI:** 10.3389/fpsyg.2022.826540

**Published:** 2022-06-02

**Authors:** Maren Rebecca Eikerling, Theresa Sophie Bloder, Maria Luisa Lorusso

**Affiliations:** ^1^Unit of Neuropsychology of Developmental Disorders, Department of Child Psychopathology, Scientific Institute IRCCS E. Medea, Bosisio Parini, Italy; ^2^Department of Psychology, University Milan-Bicocca, Milan, Italy; ^3^Faculty of Languages and Literatures, Catholic University Eichstätt-Ingolstadt, Eichstätt, Germany

**Keywords:** nonword repetition task, bilingualism, language specificity, linguistic dominance, Developmental Language Disorder

## Abstract

In bi- and monolingual children, nonword repetition tasks (NWRTs) differentiate between typically developing (TD) and children with Developmental Language Disorder (DLD). Language specificity is a crucial factor in nonword construction especially for multilingual children. While language-specific nonwords seem less artificial than non-specific nonwords, the application of language-specific phonemes may be less suitable for bilingual children who are exposed to the target language less than monolingual peers. This study evaluates the concurrent and predictive value of a novel, computerized NWRT implemented in the MuLiMi web-platform and its potential in the discrimination of bilingual children with and without DLD, investigating the role of nonwords’ language specificity. Thirty-seven children (of whom 17 had an objective risk of phonological disorders) with at least one Italian-speaking parent, living and attending kindergartens in Germany were tested with the MuLiMi NWRT and German standardized language tests. Caregivers and kindergarten teachers filled in questionnaires. Fourteen of the children were re-tested after 8–12 months. The results suggest that the new test’s concurrent and discriminative validity are good. Analysis of variance revealed highly significant differences between children with and without (an objective risk of) phonological disorders and a significant interaction between nonword specificity and risk group. Significant correlations of initial scores with follow-up scores collected after 8–12 months were also found, as well as correlations with improvements in language abilities. In conclusion, although both language-specific and language-non-specific nonword repetition can support DLD risk identification in bilingual children, language-specific stimuli appear to be particularly sensitive indicators. This is interpreted as confirming DLD children’s reduced sensitivity to frequent, familiar characteristics of the linguistic stimuli. The test’s discriminative and concurrent validity showed to be robust to various potentially influencing factors like patterns of language exposure.

## Introduction

The adequate identification of Developmental Language Disorder (DLD) in bilingual children has been described as a serious diagnostic challenge (Armon-Lotem, 2012) with continuously rising relevance as bilingualism is an increasing phenomenon worldwide (Grosjean, 2015). Previous research has identified nonword repetition tasks (NWRTs) as promising tools in the clinical differentiation between typically developing (TD) children and children with DLD not only in the monolingual population but also in bilinguals (for an overview see Schwob et al., 2021), especially since children’s NW repetition skills have been found to be associated with vocabulary (Hoover and Storkel, 2006; Farabolini et al., 2021) and grammar development (e.g., Rispens and Been, 2007). Typically, in NWRTs children are auditorily presented with a NW and are asked to immediately repeat what they just heard. Nonwords (NWs) are defined as strings of phonemes, which unlike real words do not have any meaning in the language of assessment but based on their suprasegmental, syllabic and phonological properties could potentially be real words in this language. According to Ebert (2014), the accurate repetition of a NW thus involves discrimination, encoding and production of phonemic sequences.

NWRTs are considered increasingly relevant for the identification of DLD in bilingual children as children’s phonological processing abilities can be assessed directly and—at least compared to other tasks—relatively independently of their prior vocabulary knowledge that might be reduced compared to their monolingual peers due to variations in language input and exposure. Being less subject to the influence of language experience in the language of assessment (Chiat, 2015), they would limit the disadvantage of children with reduced exposure to this language. Nonetheless, some studies have shown that they are not completely free from language-specific influences and that bilingual children perform worse on NW stimuli that were based on the phonological characteristics of their lesser known language (e.g., Boerma et al., 2015). Several studies have investigated the role of familiarity and language experience—expressed in word knowledge and/or motor routines—in repetition tasks. Dollaghan et al. (1995) found an effect of lexicality (i.e., whether a string of phonemes is a real word or not) in school-aged children. These findings were extended to younger children by Keren-Portnoy et al. (2010), who found language experience and performance to be associated with nonword vocal production at 26 months of age. Jones and Macken (2018) and Jones et al. (2020) actually found short-term verbal memory performance to be reflective of language knowledge in children at the age of 6 years. Cychosz et al. (2021) further showed that syllables in the context of a real word were more often repeated correctly than when repeated in the context of a nonword. Indeed, languages differ in terms of their suprasegmental properties, syllabic structure and complexity, and their phonological inventory, including its phonetic realization. It can be assumed that features that are not present in the repertoire of a child’s native language(s) will be more difficult to produce, whereas experience with a certain feature will be an advantage in repeating NWs that mimic this feature. NWRTs that rely on such language-specific attributes and therefore to a certain extent tap into specific knowledge of a given language have been found to be more challenging for (monolingual) children with language difficulties than for TD children (Archibald and Gathercole, 2007). Unlike TD children, children with DLD do not seem to be able to make use of prosodic and coarticulatory cues, and therefore language-specific NWs seem to have greater potential in identifying DLD. Nevertheless, they are also more sensitive to effects of language experience in bilingual children which may lead to misdiagnosis of DLD in this population.

The extant literature provides controversial evidence as to which type of NWs (i.e., language-specific vs. more neutral, i.e., non-specific stimuli) yield the optimal discriminative power for differentiating between weak language performance due to underexposure and actual language impairment in bilingual children. No effects of language experience and dominance were found on the repetition performance of bilingual TD and DLD children with either Arabic, European-Portuguese, or Turkish as their L1 (family or heritage language) who used the French and the German versions of the LITMUS NWRT (De Almeida et al., 2017; Tuller et al., 2018)—not distinguishing between language-specific and language-non-specific items. Dos Santos and Ferré (2016) did not find any significant difference when comparing monolingual and bilingual children’s repetition performance, irrespective of syllable complexity. Similarly, in the study of Abed Ibrahim and Hamann (2017), no differences emerged when comparing monolingual TD and bilingual TD children as well as monolingual DLD and bilingual DLD children, and this was true for both language-specific and non-specific NWs. Yet, the mono- and bilingual children with DLD did perform worse than the TD children, and this was more evident for the language-specific than for the non-specific NWs. Thus, there were no differences between monolingual and bilingual children, but there were differences between TD and DLD, and these depended on language-specific more than on language-non-specific NWs (for language-non-specific NWs, no significant differences emerged between TD and DLD). Additionally, within the several LITMUS NWRTs, language-specific and non-specific items were created following similar criteria, with the only exception that for the language-specific NWs a few additional phonemes were used (e.g., Grimm and Hübner, 2017). So, especially when language-non-specific items were presented with the prosody of the language of assessment, language-specific and language-non-specific items may have been too similar to show significant differences in children’s repetition performance.

The number of conclusions to be drawn from previous research is limited, especially because findings on the relationships and interactions between language experience and repetition performance on language-specific NWs are mixed. In this study we compare NWs that take into account the characteristics of both languages and NWs that are relatively free (or freer) from such language-specific attributes in order to establish which NWs are more useful (discriminant) for the language assessment of bilingual children.

Indeed, a prominent issue concerns the need or usefulness of administering NWRTs in the L1, in the L2 or in both languages for the diagnosis of DLD. Gutiérrez-Clellen and Simon-Cereijido (2010) suggest that assessment in only the child’s dominant language may lead to the underidentification of DLD and highlight the importance of bilingual approaches (i.e., assessing repetition performance in both languages) in order to increase identification accuracy. Summers et al. (2010) further point out that experience-effects may not be limited to one language and may be visible in different NWRTs regardless of the language they reflect (see also Gibson et al., 2015). Windsor et al. (2010) found that bilingual Spanish-English TD children performed better than monolingual DLD children on the English NWRT (see also Kohnert et al., 2006), confirming that NW repetition can reliably highlight the presence of a language disorder irrespective of language exposure. Similarly, Ahufinger et al. (2021) found that although bilingual children generally show worse NW repetition performance than their monolingual peers, NWRTs represent valid tools to distinguish DLD from TD across a range of linguistically diverse populations. Armon-Lotem and Meir (2016) further found that both L1 and L2 NWRTs effectively discriminate bilingual children with and without DLD, especially when bilingual cut-off points are applied. Taken together these findings suggest that even when applying a bilingual approach by assessing NW repetition performance in both a child’s languages, performance may depend on the level of specificity of a given NWRT, with more specific NWRTs showing greater influence of experience.

Thordardottir and Juliusdottir (2013) for Icelandic as L2 (societal or second language), as well as Lee et al. (2013) for Korean as L1, showed that bilinguals’ performance on a NWRT that followed the phonotactic constraints of the language is independent of when and for how long they had been exposed to it. Also Farabolini et al. (2021) failed to find any significant correlations between bilingual children’s Italian (L2) NW repetition performance and language exposure. These studies suggest that performance on language-specific NWRTs is in fact independent of specific knowledge of the structure of a particular language. Similarly, Core et al. (2017) found no association between their bilingual Spanish-English participants’ language experience and their performance on an English and a Spanish NWRT. However, Parra et al. (2011) found positive correlations between children’s repetition performance and language input in English (L2) for English NWs [but not for Spanish (L1) NWs]. Since the participants in Core et al. (2017) were older than the participants included in the study by Parra et al. (2011) (mean age 30 vs. 22 months), it is possible that the effects of language experience are more observable in the early stages of language exposure. Furthermore, Engel de Abreu (2011) found better performance in a Luxembourgish NWRT for monolingual compared to simultaneous bilingual children, but this effect disappeared once children’s lexical knowledge was taken into account, suggesting that the difference in repetition performance was moderated by children’s language experience.

This heterogeneity in findings may be explained by the great variability in methodological approaches that were applied in these studies, such as differences in the construction of the NWRTs, the characteristics of the NWs, the targeted language of assessment (the children’s L2 or L1) and the children’s age and patterns of language dominance.

Variations in results concerning the influence of language-specific knowledge on children’s NW repetition performance may also depend on how language experience was defined and measured across the different studies. Generally, gathering children’s language background information through parents’ and/or teachers’ reports (*via* questionnaires) has proven to be a sound method (Pua et al., 2017). However, adequate quantification of language experience has been proven to be difficult and there is no univocal, best-practice approach (Hoff, 2020). Age of onset of exposure (e.g., Thordardottir and Juliusdottir, 2013), current exposure (e.g., Sharp and Gathercole, 2013), and cumulative exposure (e.g., Thordardottir, 2017) have previously been used to measure the influence of language experience on NW repetition performance. However, as previously pointed out by Farabolini et al. (2021), up to this point, no study has considered the effects of children’s active language use (i.e., the relative amount of children’s production in either of their languages) on children’s accuracy in NW repetition. When comparing children’s repetition of real words and NWs, Dispaldro et al. (2013) found significantly reduced scores in DLD children for NW repetition accuracy compared to real-word repetition accuracy. This may suggest that children, when repeating real words, can rely on well-learned production routines (see also Keren-Portnoy et al., 2010). In fact, these routines may be more beneficial to the repetition of language-specific than non-specific NWs as higher phonological frequency and/or higher word-likeness (i.e., the resemblance between a NW and a real word) have been found to result in in higher NW repetition accuracy (Munson et al., 2005; Ahufinger et al., 2021).

Based on the above reviewed evidence, the present study aims to follow up on the question concerning which type of NWs is most informative about children’s language ability status. We consider both (1) language-specific (LS) items that take children’s language experience/dominance into account and (2) language-non-specific (LNS) items that are intended to assess children’s language abilities regardless of their language background. In order to avoid circular procedures, we will address diagnostic accuracy, taking into account information deriving from alternative tests of phonological ability and information collected through parental and kindergarten teachers’ questionnaires, including active use of language in addition to passive exposure. Finally, the goal of this study is to assess the validity of the “MultiMind Nonword Repetition Task,” which is part of a web-platform (called MuLiMi, Eikerling et al., 2022), especially developed and designed to allow any examiner, regardless of his/her own language background, to assess children’s language abilities in both their L1 and L2 through fully automatized, highly standardized tests and procedures.

The validity of the NWRT will be analyzed through:

- The correlations of children’s NW repetition scores with other standardized phonological scores, as well as with parents’ and teachers’ judgments of the children’s language abilities (concurrent validity).
- The differences emerging between the groups of children with and without an objective risk of having DLD (in general or more specifically affecting phonological skills) based on pre-existing diagnoses and/or standardized language tests scores (discriminative validity).
- The correlations between NWRT scores at T1 and follow-up (T2) scores (predictive validity).

In order to identify the type or the types of NWs that have the best discriminative power we pose the following research questions (RQs):

- *RQ1*: Is MuLiMi NWRT repetition accuracy valid and reliable in the identification of DLD (general risk of DLD or more specifically, risk of a phonological disorder) as assessed by children’s risk status based on German standardized test scores? If yes, is performance in repeating LS items or LNS items more discriminant? And are accuracy scores on the (various types of) NWRT in accordance with parental questionnaires and kindergarten teachers’ subjective ratings?
- *RQ2*: Are there any advantages in using a bilingual NWRT over a monolingual one?
- *RQ3*: Is children’s language dominance correlated with children’s NW repetition accuracy and does language experience (dominance and exposure) influence improvement in NW repetition accuracy?
- *RQ4*: Does NW repetition accuracy at T1 predict improvement in overall language performance?

## Materials and Methods

### Participants

Thirty-nine simultaneous or early-sequential bilingual Italian-German speaking children aged from 3;10 to 6;1 years (age in months: *M* = 59.05, SD = 8.56) participated in this study. For the purpose of this study, two participants were excluded because of contradictory information in their parental questionnaires concerning whether or not they had an official diagnosis of DLD. At the time of testing, out of the 37 children, seven had already been diagnosed with DLD by German Speech and Language Therapists and were receiving treatment in Germany, 13 were considered TD, and 17 had neither been diagnosed nor treated for DLD but were identified as being at-risk for language impairment because they scored below a *t*-score of 40 in at least one of the standardized tests applied in this study (see section “German Standardized Tests”). Due to the phonological nature of the NWRT, a more specific type of “phonological risk” was identified for appropriate comparisons. This group comprised children who scored below a *t*-score of 35 in the German standardized NWRT (Mottier-test). According to this criterion, 10 children did, and 27 children did not, present with phonological risk. When participating in this study, all children were living in Germany. They all had at least one native Italian-speaking parent and were exposed to Italian—even though to varying degrees—on a daily basis. Participants with two Italian-speaking parents had been exposed to the German language for at least 2 years. While eight of the children were attending a German kindergarten, 31 were enrolled in a bilingual Italian-German kindergarten program. A subset of the children (*n* = 14; three TD, five DLD, six at-risk) participated in a follow-up study (T2) that occurred 8–14 months after the first evaluation (T1) repeating both standardized and experimental tests. Participant recruitment took place through either kindergartens or Speech and Language Therapy clinics. The study was approved by the institutes’ Ethical Committees and all parents signed informed consent according to the Declaration of Helsinki.

### Assessment Tools

All participants were tested on a whole battery of assessment tools, including three standardized German tests (some of which also had bilingual norms) and an additional experimental protocol constituting a novel, web-based set of screening tasks for the assessment of risk of DLD (under validation). The Italian-German NWRT that is reported in this study is part of this experimental battery. Furthermore, caregivers were asked to complete a language background questionnaire providing information about their children’s language experience and exposure patterns and an extensive questionnaire on pregnancy, general and language development. Finally, kindergarten teachers were asked to complete a short survey on the children’s language skills across linguistic areas.

#### MuLiMi NWRT

The NWRT included in the MuLiMi screening battery consists of a total of 21 NWs (see Supplementary Table D). It includes two sets of LS NWs (one set that is specific with respect to phonotactic constraints of Italian, LS Italian, *n* = 6; the other set is specific with respect to German ones, LS German, *n* = 6), recorded by expert native speakers of the respective language, and one set of LNS NWs (five of the items were recorded by an expert native speaker of Italian, while the other four items were recorded by an expert native speaker of German). To make sure that the LS NWs complied with the phonotactic constraints of the respective language, phonemes and consonant clusters specific to each language were identified and used (for German: Orzechowska and Wiese, 2015; for Italian: Baroni, 2012). To create LNS NWs, only phonemes present in both languages were used (differences in the phonetic realization of these phonemes, e.g., voicing for stop consonants were considered tolerable). As opposed to the LS items, no consonant clusters were included in the LNS NWs. Because of this the LS items could be considered more complex than the LNS items (see also Dos Santos and Ferré, 2016). To make sure that errors in children’s repetition were not due to speech production difficulties, late acquired phonemes (based on the phonological development trajectories of monolingual TD children for German see Fox, 2000; for Italian see Bortolini, 1995) were not included in the NWs. During the task, LS items are presented with the prosodic features of the respective language, whereas LNS items are presented with flat, neutral prosody (carefully avoiding placing lexical stress on any specific syllable in the NW, see Mottier, 1951; Chiat, 2015) so as not to reflect any pattern of lexical prosody that could be typical of a specific language. The selection of NWs included in the NWRT of the Italian-German screening from the initial set of 55 items (21 LS Italian, 18 LS German, and 16 LNS NWs) was based on a 2-step rating procedure (Bloder, Eikerling, and Lorusso, submitted): first, after auditory presentation, monolingual adult native speakers of the respective language were asked to repeat each NW and rate its L1-alikeness in the rater’s language and pronounceability on a scale from 1 to 5. Based on these repetition, L1-alikeness and pronounceability scores, a subset of NWs was selected that was rated again for pronounceability and language-specificity by a new group of adult native speakers using an online questionnaire (Qualtrics, 2020). This process determined the final selection of NWs implemented on the screening platform MuLiMi and presented *via* a computer. NW stimuli were presented in random order so as to avoid a potential habituation effect, i.e., preventing the child from accustoming him/herself to the phonological and prosodic features of one of his/her languages before switching to the repetition of stimuli of a different kind. In order to help children focus their attention on listening and repeating the NWs, an illustration depicting a space scenery was displayed on the computer screen. One of the elements displayed in this picture automatically changed after each NW repetition. A bilingual Italian-German speaker judged children’s repetition accuracy on the whole-word level. Children’s repetition attempts were either scored as correct (1; if judged by the examiner to fully match the target) or incorrect (0, if children’s realization of the NW deviated from the target by one or more phonemes). As noted before, variations in accent or in the phonetic realization of single phonemes depending on language-specific features (e.g., variations in the realization of /r/ or /p/ according to the phonetic features of German or Italian) were considered acceptable as long as the phoneme was clearly identifiable. The raw scores obtained in the MuLiMi NWRT reflect the number of NWs that were correctly repeated by each child (e.g., Chiat and Polišenská, 2016). Finally, the percentage of overall accuracy was recorded. Inter-rater reliability (IRR) was calculated from two further examiners (one native German speaker and one native Italian speaker) who independently assigned scores to children’s NW repetitions for a subset of 29 children (all seven DLD children and 22 of the non-DLD children). For IRR (based on three raters), Cronbach’s Alpha was *α* > 0.70 for all NWs. Internal consistency as expressed by Cronbach Alpha was *α* = 0.903.

#### Crosslinguistic-Lexical Task (Receptive Lexicon)

A further LITMUS-task, *the Crosslinguistic-Lexical Tasks* (CLTs; Haman et al., 2017), was used in Italian and German. In the noun and verb comprehension subtests, after the automatic auditory presentation of a pre-recorded (native speaker) noun or verb, children are asked to indicate the target picture among four colored line drawings. Each of the subtests contains 32 items (64 items per language, 128 in total). An example from the German noun subtest is “Zeige mir ‘Gürtel’” [Show me ‘belt’], an example from the Italian verb comprehension subtest is “Chi sta giocando a golf?” [Who is playing golf?].

#### German Standardized Tests

##### Mottier-Test

In order to obtain a standardized phonological score, a German NWRT, the Mottier-test (original version created by Mottier, 1951; norms by Risse and Kiese-Himmel, 2009) was used. It consists of 30 NWs that range from two (e.g., “rela”) to six syllables (e.g., “bigadonafera”) and include only simple CV structures. NWs were pre-recorded by a native speaker of German and presented one by one *via* a computer, at the rate of one syllable per second. Children are asked to immediately repeat each NW after presentation. Repetition accuracy was assessed by the examiner and one point was assigned to every correctly repeated NW. The maximum score is 30.

##### LiSe-DaZ

Children’s morpho-syntactic abilities in German were assessed using the elicited production task of the Linguistische Sprachstandserhebung Deutsch als Zweitsprache (LiSe-DaZ; Schulz and Tracy, 2011) that provides norms for monolingual German children as well as for second language learners of German. The production task of the LiSe-DaZ addresses several areas of German morpho-syntax. Scores are derived from the analysis of a sample of spontaneous speech elicited through a picture story and semi-structured storytelling interview. Due to their particular relevance for NWRTs, the domains of verb placement and subject-verb-agreement were investigated in detail in this study. The pictures from the test’s storytelling book are presented and children’s reactions to the pre-defined elicitation questions asked by the examiner (e.g., “Was fragt Lise?” [What is Lise asking (them)?]) are transcribed and then analyzed. *Production of complex sentences* & *verb placement* is judged on a scale ranging from 1, indicating an utterance of a single word only, to 4, indicating the use of embedded sentences with the verb in sentence-final position in a subordinate clause. For instance, the question “Warum macht der Hund so ein trauriges Gesicht?” [Why is the dog making such a sad face?] is used to elicit children’s production of a subordinate clause. A common, correct level 4 response is “Weil er in der Mülltonne ist” [Because he is (caught) in the garbage can]. By contrast, an utterance such as “Weil ist traurig” with the verb in second position is considered level 3. A level is considered mastered if the child produces at least three utterances corresponding to that level throughout the entire test. As for *Subject-verb-agreement*, scores are calculated by first identifying all utterances that contain a subject and a verb and in a next step counting all the utterances with correct subject-verb agreement. Finally, the ratio between the number of occasions for subject-verb-agreement and the correctly realized instances is calculated. For instance, a common, correct response to the question elicitation “Was passiert hier?” [What is happening (in this picture)?] is “Die (Kinder) spielen Fußball.” [They are playing football.] By comparison an utterance such as “Spielen Fußball” is not included in the analysis as it is missing the subject. The maximum score is 1.0, children’s performance again is represented on a four-point scale.

##### Peabody Picture Vocabulary Test

The German adaptation of the Peabody Picture Vocabulary Test (PPVT-4; Lenhard et al., 2015) was used for the assessment of children’s receptive lexical abilities in German. It consists of 19 blocks each containing 12 items of increasing complexity and decreasing frequency. In this test, the child first listens to a pre-recorded word (natural voice, native speaker of German) and is then asked to choose the corresponding picture out of four possible answers. The test is aborted if a child fails in eight consecutive trials within one block. Scores are obtained by assigning one point for each time children’s selection of the picture did not match the word that was presented. The total number of errors is then subtracted from the number of the last item before the test was finished/aborted.

##### Colored Progressive Matrices

The German adaptation of Raven’s Colored Progressive Matrices (CPM, Bulheller and Häcker, 2001) was used for the assessment of children’s nonverbal intelligence. Performance is expressed as *t*-scores.

#### Questionnaires

##### QUIR-DC (Questionario per l’Individuazione del Rischio—Disturbi della Comunicazione—Questionnaire for the Identification of Risk for Communication Disorders)

The Italian version of the QUIR-DC (Lorusso and Dolzadelli, 2016, designed for clinical use on the IRCCS Medea online platform) was translated into German and adapted as a pen-and-paper questionnaire that was filled in by children’s parents. The questionnaire includes a total of 96 questions. Besides questions on anagraphical data and information on the child’s language background, parents’ responses correspond to scores used to compose a global and a risk score, respectively. Precisely, positive scores contribute to a general score (GS) expressing the level of development, whereas negative scores contribute to the risk score (RS) expressing the probabilistic risk that the child has a developmental delay or disorder (distinguishing among a general delay and more specific language delays, including a phonological score). Finally, the family global language input score (FIGS) is calculated, expressing the quality of language input in the child’s L2. Parents could choose between the Italian and German versions of the questionnaire. Data was entered and scored automatically using the Formfacade web application (Formfacade, 2021).

##### Language Background Questionnaire

A detailed pen-and-paper questionnaire (Bloder, Rinker, and Shafer, in prep.) was used to assess children’s language background. Depending on the families’ preference, the questionnaire was provided either in German or in Italian. The questionnaire included 34 questions concerning what language(s) children hear and speak on a daily basis. Children’s main caregivers were asked to use a seven-point scale using a combination of frequency adverbs and a percentage scale to estimate the proportion of their children’s Italian compared to German exposure in different contexts (one example for language input in the home: “What language(s) does the child hear from his/her mother” (1) only German; 100% German, 0% Italian, (2) predominantly German, hardly any Italian; 90% German, 10% Italian, (3) mostly German, sometimes Italian; 75% German, 25% Italian, (4) the same amount of German and Italian; 50% German, 50% Italian, (5) sometimes German, mostly Italian; 25% German, 75% Italian, (6) hardly any German, predominantly Italian; 10% German, 90% Italian, and (7) only Italian; 0% German, 100% Italian). Based on this information, an individual input (i.e., in regard to the language(s) the child hears on a daily basis) and output (i.e., in regard to the language(s) the child speaks on a daily basis) score were calculated for each language. The values obtained reflect the children’s current language experiences at the time of their participation in this study.

##### Kindergarten Teacher Questionnaire

A further questionnaire was completed by the child participant’s kindergarten teacher concerning the presence of difficulties in the domains of phonology, lexicon, morphosyntax and pragmatics. Each domain was judged according to a four-level scale (no difficulties, mild/moderate/severe difficulties).

### Procedure

Each child was tested individually in a quiet room by well-trained researchers either in one of the kindergartens or one of the Speech and Language Therapy clinics where they were recruited. In the few cases where this was not possible due to COVID-19 contact restrictions, children were tested in a quiet room in their homes. The test battery was administered in two separate sessions lasting between 40 and 50 min each. The NWRT was carried out using a Lenovo laptop, model YOGA 720-15IKB under the Windows 10 Pro operating system. The online screening platform MuLiMi was accessed *via* the Mozilla Firefox web browser. For a subset of children (*n* = 14), follow-up (T2) data was collected between 8 and 14 months after the initial evaluation (T1). Parents and teachers’ questionnaires were filled individually on printed forms and returned to the researchers after a few days.

### Data Analysis

Children were assigned to one of three groups (clinical/risk status: TD, at-risk of DLD, DLD) based on the information about a pre-existing diagnosis of DLD (DLD group) and whether they had obtained a t-score below 40 in at least one of the standardized tests applied in the study’s test protocol but without a pre-existing diagnosis of DLD (at-risk group). A further, more specific criterion for classifying children at-risk for phonological problems was applied dividing the group with respect to a cut-off of 35 (i.e., −1.5 SDs) for the t-score in the Mottier test (below or at cut-off: phonological risk).

A variable expressing linguistic dominance was created based on parental reports, estimating the average number of hours each child is exposed to/actively speaks Italian during a regular week. Then, the number of hours was converted into a percentage in order to express the ratio between children’s weekly language input and output in Italian vs. German. A compound score for language dominance in which the input and output scores of both languages were merged was created based on the following equation:


$$Language\;Dominance=\frac{\left(\begin{array}{c} \left(InputIT-InputGER\right)\\ +\left(OutputIT-OutputGER\right) \end{array}\right)}{2}$$

Based on the resulting score, children’s language dominance was assigned. The variable displaying dominance ranges from −1 (German dominant) to 1 (Italian dominant). Children with a score of −1 to −0.16 were considered German dominant; −0.15 to 0.15 balanced; 0.16 to 1 Italian dominant.

Data were analyzed by means of IBM SPSS Statistics v.26. The association between children’s NW repetition performance and their linguistic profile was assessed through partial correlation analyses, controlling for children’s language dominance, age (in months), and nonverbal intelligence (CPM *t*-scores). Point-biserial correlations were computed for the association with dichotomous variables (presence/absence of DLD or phonological risk), whereas Spearman’s correlations were used in the case of three- or four-levels variables (clinical risk status, LiSe-DaZ verb placement & subject-verb-agreement). In all other cases Pearson’s correlations were computed, under the condition that the variables’ distributions satisfied all requirements for such analyses. Nonparametric tests were used for the comparisons of small groups (namely, when the DLD group, comprising seven children, was compared to other groups) or for non-continuous variables. Repeated-measures ANOVAS were performed on continuous, close-to-normally distributed variables (the scores obtained on various subsets of the NWRT) with Specificity (LS vs. LNS) and (after excluding LNS NWs) Language (LS-Italian vs. LS-German) as within-subject factors and Phonological risk (with vs. without) as a between-subjects factor. The effects of dominance as a covariate were also assessed. Nonverbal intelligence and age were added as covariates (or controlled for in partial correlation analyses) whenever they had been found to correlate with the variables under exam.

For the subset of 14 children who participated in the follow-up study, a NWRT improvement score was calculated by subtracting their repetition performance at T1 from their scores at T2. To preliminarily assess the predictivity of the MuLiMi NWRT, nonparametric correlation analyses were computed between children’s repetition performance at T1 and their improvement in the various NW subcategories.

No Bonferroni correction was applied when the analysis responded to *a priori* hypotheses, nor when it involved a set of mutually correlated variables. In all other cases, Bonferroni correction and the value of alpha are specified in the results section.

## Results

### Description of the Sample

Table 1 provides an overview of the children in accordance with their clinical/risk status (TD, at-risk, DLD). Further descriptive information is provided in the Supplementary Table A. Nonparametric tests were used for group comparisons considering the small size of the DLD group and the ordinal nature of some of the Lise-DaZ scales. Across the three groups, children’s age did not differ significantly (Kruskal–Wallis, *p* > 0.05). While the TD children leaned more towards the German-dominant end of the continuum compared to the DLD children who as a group were rather dominant in Italian, the children in the at-risk group represented a greater variety of experience patterns. Even though the difference in dominance patterns did not reach significance across the three groups (Kruskal–Wallis, *p* = 0.066), pairwise comparisons showed a significant difference in language dominance between the TD and the DLD group [*t*(16.584) = −3.621, *p* = 0.002], whereas for the TD vs. at-risk group and the at-risk vs. DLD group no significant differences emerged (*p_s_* > 0.05).

**Table 1:** Description of the sample at T1 grouped according to clinical/risk status.

	TD (*n* = 13)	At-risk (*n* = 17)	DLD (*n* = 7)
Age (in months)	*M* = 60.83, SD = 10.69	*M* = 58.47, SD = 8.46	*M* = 57.86, SD = 6.54
Dominance (TD: *n* = 12)	*M* = −0.246, SD = 0.323 German dominant: 53.85% Italian dominant: 7.69% Balanced: 38.46%	*M* = −0.099, SD = 0.567 German dominant: 47.06% Italian dominant: 23.53% Balanced: 29.41%	*M* = 0.201, SD = 0.214 German dominant: 0% Italian dominant: 71.43% Balanced: 28.57%
Mottier (raw scores, max. 30)	*M* = 15.08, SD = 3.62	*M* = 9.47, SD = 4.65	*M* = 3.14, SD = 3.89
CPM (*t*-scores)	*M* = 56.10, SD = 15.45	*M* = 44.90, SD = 9.23	*M* = 38.80, SD = 6.96
LiSe-DaZ verb placement (1–4)	*M* = 4.00, SD = 0.00	*M* = 3.0, SD = 1.55	*M* = 1.75, SD = 1.50
LiSe-DaZ subj.-verb-agr.^1^ (1–4)	*M* = 4.00, SD = 0.00	*M* = 2.73, SD = 1.68	*M* = 0.50, SD = 0.58
PPVT-4 (raw scores)	*M* = 101.71, SD = 20.43	*M* = 85.45, SD = 36.36	*M* = 42.00, SD = 33.73

1 LiSe-DaZ subject-verb-agreement.

In both the CPM and the Mottier-test, the TD children scored highest, followed by at-risk and then DLD children (see Table 1 for a detailed overview). A Kruskal–Wallis H test showed a statistically significant difference across the three groups (TD, at-risk, and DLD) in CPM scores [*χ^2^*(2) = 11.232, *p* = 0.004, with a mean rank of 26.46 for TD, 16.69 for at-risk and 10.50 for DLD children]. A Mann–Whitney-*U*-Test confirmed significant differences for TD vs. at-risk children (*U* = 51.50, *Z* = −2.474, *p* = 0.013) and TD vs. DLD children (*U* = 7.50, *Z* = −3.013, *p* = 0.003) but not for at-risk vs. DLD children (*p* > 0.05).

Performance (raw scores) in the Mottier-test was normally distributed in the sample. Running pairwise comparisons, significant differences emerged in the Mottier-test raw scores for TD vs. at-risk children [*t*(28) = 3.590, *p* = 0.001], at-risk vs. DLD children [*t*(22) = 3.244, *p* = 0.004] and TD vs. DLD children [*t*(18) = 7.188, *p* < 0.001].

Table 2 provides an overview of the sample grouped according to phonological risk status (find further descriptives in the Supplementary Table B). The results described for clinical/at-risk status (Table 1) were substantially confirmed, except for language dominance which does not significantly differ between the two groups.

**Table 2:** Description of the sample at T1 grouped according to phonological risk status, and t-test comparisons.

	No phonological risk (*n* = 27)	Phonological risk (*n* = 10)	Group comparison
Age (in months)	*M* = 59.12, SD = 9.72	*M* = 59.20, SD = 6.52	*t*(35) = 0.008, *p* > 0.05
Dominance (no phonological risk: *n* = 26)	*M* = −0.108, SD = 0.435 German dominant: 44.44% Italian dominant: 18.52% Balanced: 37.04%	*M* = −0.042, SD = 0.548 German dominant: 30.00% Italian dominant: 20.00% Balanced: 50.00%	*t*(34) = −0.378, *p* > 0.05
Mottier (raw scores, max. 30)	*M* = 12.81, SD = 4.53	*M* = 3.20, SD = 2.25	*t*(35) = 6.524, *p* < 0.001
CPM (*t*-scores)	*M* = 51.53, SD = 13.35	*M* = 37.91, SD = 5.97	*t*(35) = 3.083, *p* = 0.004

No significant associations between age and the NWRT emerged for any of the scales (*N* = 37, *r_s_* < 0.251, *p_s_* > 0.135), except for LS-German (*r* = 0.414, *p* = 0.011). Children’s NWRT scores across scales correlated significantly with the raw scores obtained in the CPM (nonverbal intelligence; *N* = 37, *r_s_* ranging from 0.338 to 0.450, *p_s_* ≤ 0.041), but when controlling for age, these effects disappear for LS-German (*r* = 0.159, *p* = 0.353) and, consequently, LStot (*r* = 0.292, *p* = 0.083).

### NW Repetition and Children’s Risk Status

**Figure 1:**
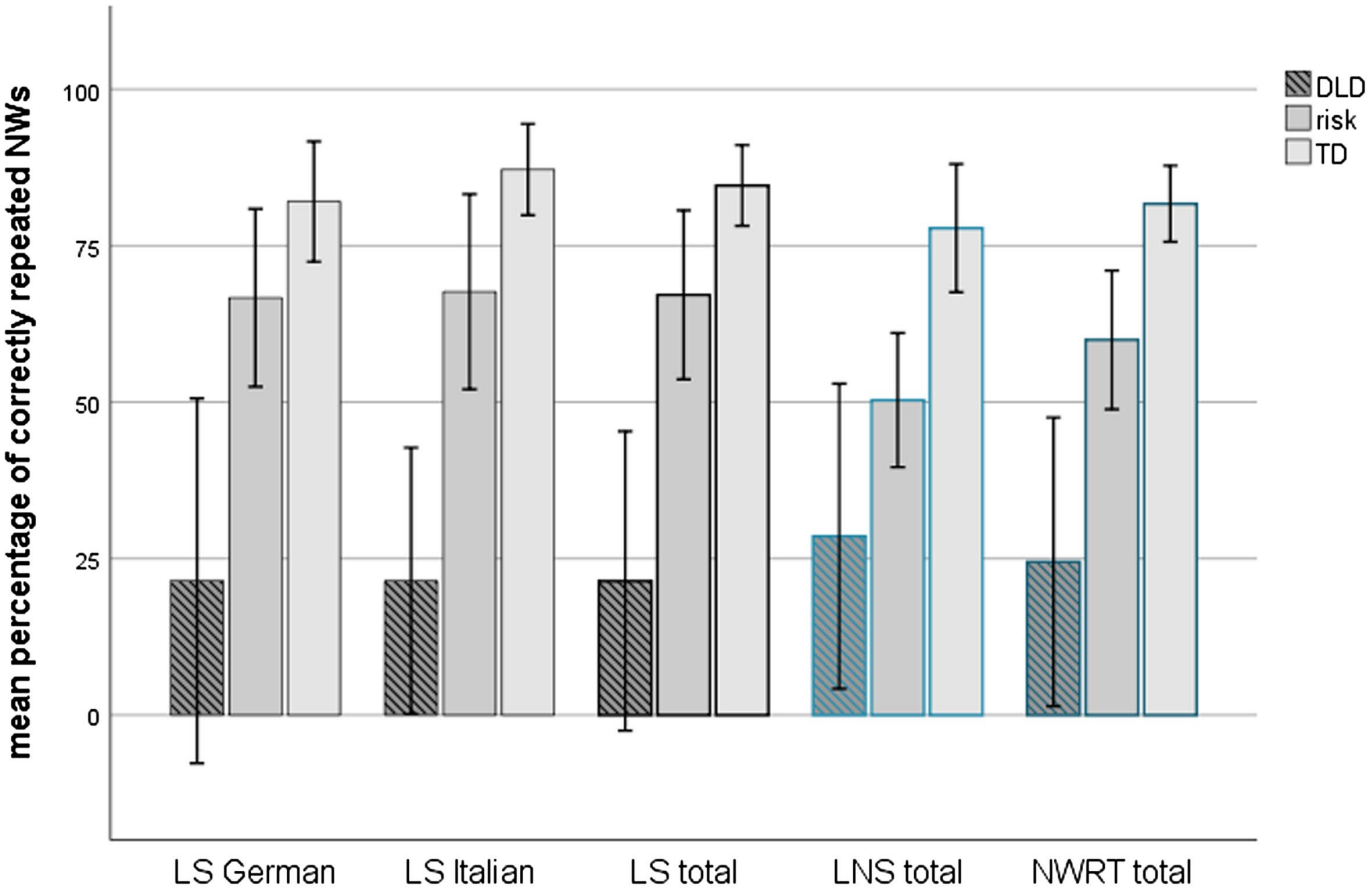
Mean percentage of correctly repeated NWs for each MuLiMi NWRT scale according to clinical/risk status at T1. Error bars represent 1 SD. TD: *n* = 13; at-risk: *n* = 17; and DLD: *n* = 7.

As shown in Figure 1, children’s NW repetition performance in all subcategories differed between the three clinical/risk status groups. A non-parametric Kruskal–Wallis test revealed significant differences (*χ*^2^ between 12.685 and 19.686, *p*_s_ ≤ 0.002) between the three groups for all five comparisons, and *post-hoc* tests confirmed that differences between TD and DLD children were always significant (Mann–Whitney *U* test, *Z* ≥ 3.259, *p* < 0.001) while differences between TD and at-risk children were always significant (*Z* ≥ −2.132, *p*_s_ ≤ 0.035) except for the single lists of LS NWs in Italian and in German (*p*_s_ ≥ 0.053). Similarly, differences between at-risk children and children with DLD were all significant (*p*_s_ ≤ 0.007) except for LNS NWs (Z = −1.804, *p* = 0.075). This general pattern was also confirmed by the significant correlations (Spearman) found between NWRT in all scales and the three-levels classification of clinical risk: *rho_s_* (*n* = 37; between 0.559 and 0.739, *p*_s_ < 0.001). Across all subcategories, TD children achieved the highest scores, followed by at-risk and DLD children.

A further analysis was performed on the scores obtained on the various types of NWs according to the children’s phonological risk (*n* = 10 with, *n* = 27 without phonological risk). An independent sample *t*-test confirmed that across all subcategories NWRT performance was significantly worse for children with, compared to children without phonological risk (see Table 3).

**Table 3:** Mean percentages of correctly repeated NW for all MuLiMi NWRT scales according to children’s phonological risk status at T1, and *t*-test comparisons.

	No phonological risk (*n* = 27)	Phonological risk (*n* = 10)	*t*-test
LS-Italian	*M* = 82.70, SD = 14.96	*M* = 20.01, SD = 23.31	*t*(35) = 9.681, *p* < 0.001
LS-German	*M* = 76.54, SD = 19.20	*M* = 28.35, SD = 36.05	*t*(35) = 5.279, *p* < 0.001
LS-total	*M* = 79.62, SD = 13.74	*M* = 24.14, SD = 28.72	*t*(35) = 7.984, *p* < 0.001
LNS-total	*M* = 65.03, SD = 21.95	*M* = 31.11, SD = 25.03	*t*(35) = 4.022, *p* < 0.001
NWRT-total	*M* = 73.37, SD = 14.73	*M* = 27.13, SD = 25.98	*t*(35) = 6.828, *p* < 0.001

**Figure 2:**
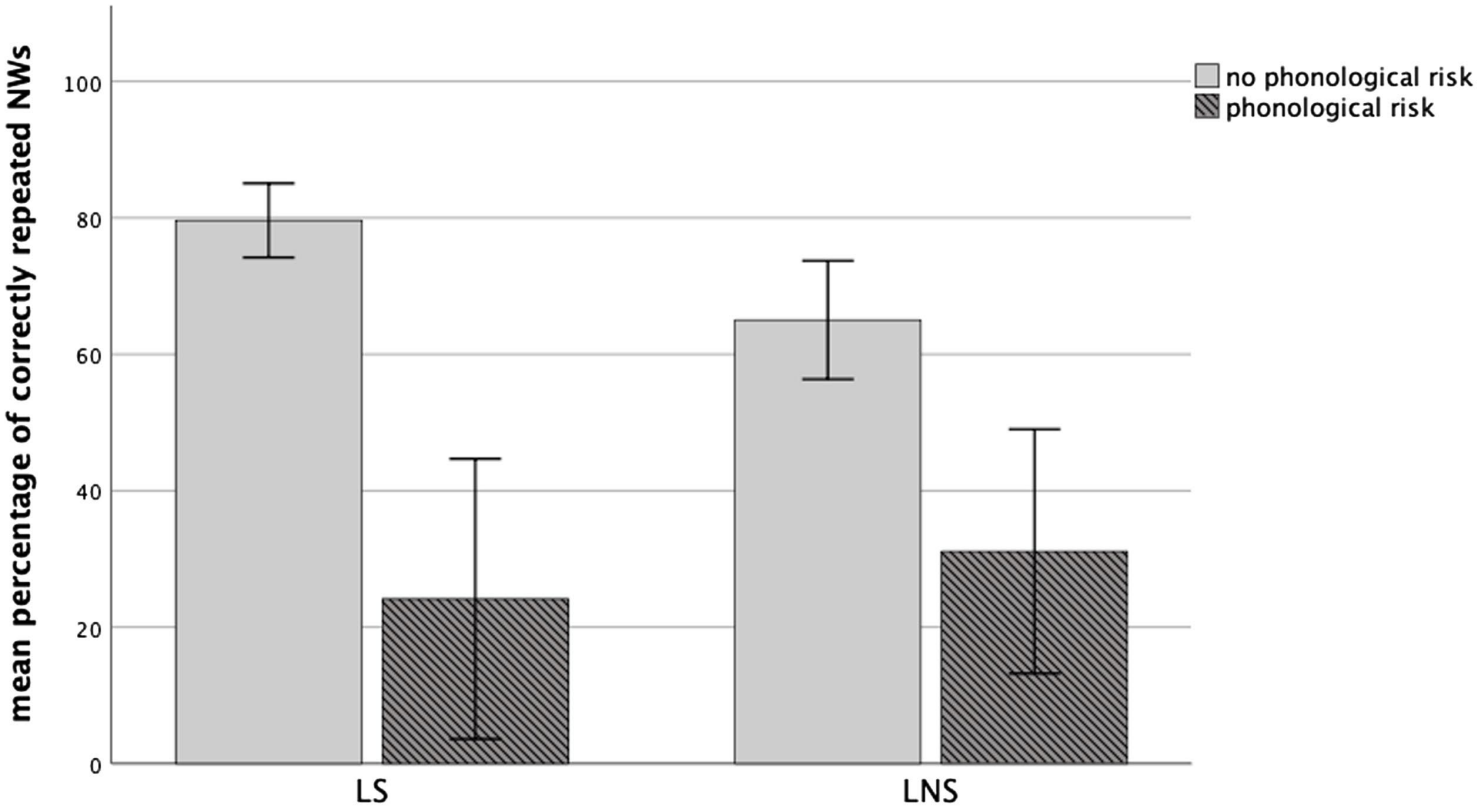
Children’s mean percentage of correctly repeated LS vs. LNS NWs in the MuLiMi NWRT according to their phonological risk. Error bars represent 1 SD. No phonological risk: *n* = 27; phonological risk: *n* = 10.

Furthermore, a repeated-measures ANOVA with NW Specificity (LS vs. LNS) as within-subject factors and Phonological risk (with vs. without) as a between-subjects factor and dominance, nonverbal intelligence and age as covariates was performed. There was no significant main effect for Specificity (*p* > 0.05) but a significant between-subjects effect for Phonological risk status [*F*(1, 31) = 28.174, *p* < 0.001, *η*^2^ = 0.476]. However, there was a significant interaction effect between NW Specificity and children’s Phonological risk status [*F*(1, 31) = 13.053, *p* = 0.001, *η*^2^ = 0.296]. *Post-hoc* tests showed that the NW repetition performance was significantly different for children with and without Phonological risk for both types of NWs [LS NWs: *t*(35) = 7.984, *p* = <0.001; LNS NWs: *t*(35) = 4.022, *p* < 0.001]. The repetition of LS NWs was significantly better than repetition of LNS NWs for the children without risk [*t*(26) = 3.801, *p* = 0.001], whereas no significant difference emerged for the children with Phonological risk, who even performed (non-significantly) better with LNS than with LS NWs [*t*(9) = −1.366, *p* = 0.205; see Figure 2]. A significant effect emerged for the covariate age [*F*(1, 31) = 4.409, *p* = 0.044, *η^2^* = 0.125], but not for nonverbal intelligence and language dominance (*p_s_* > 0.05). No significant interaction between NW Specificity and children’s language dominance (*p* > 0.05) emerged either. Furthermore, the covariates did not show any significant interaction with Phonological risk.

**Figure 3:**
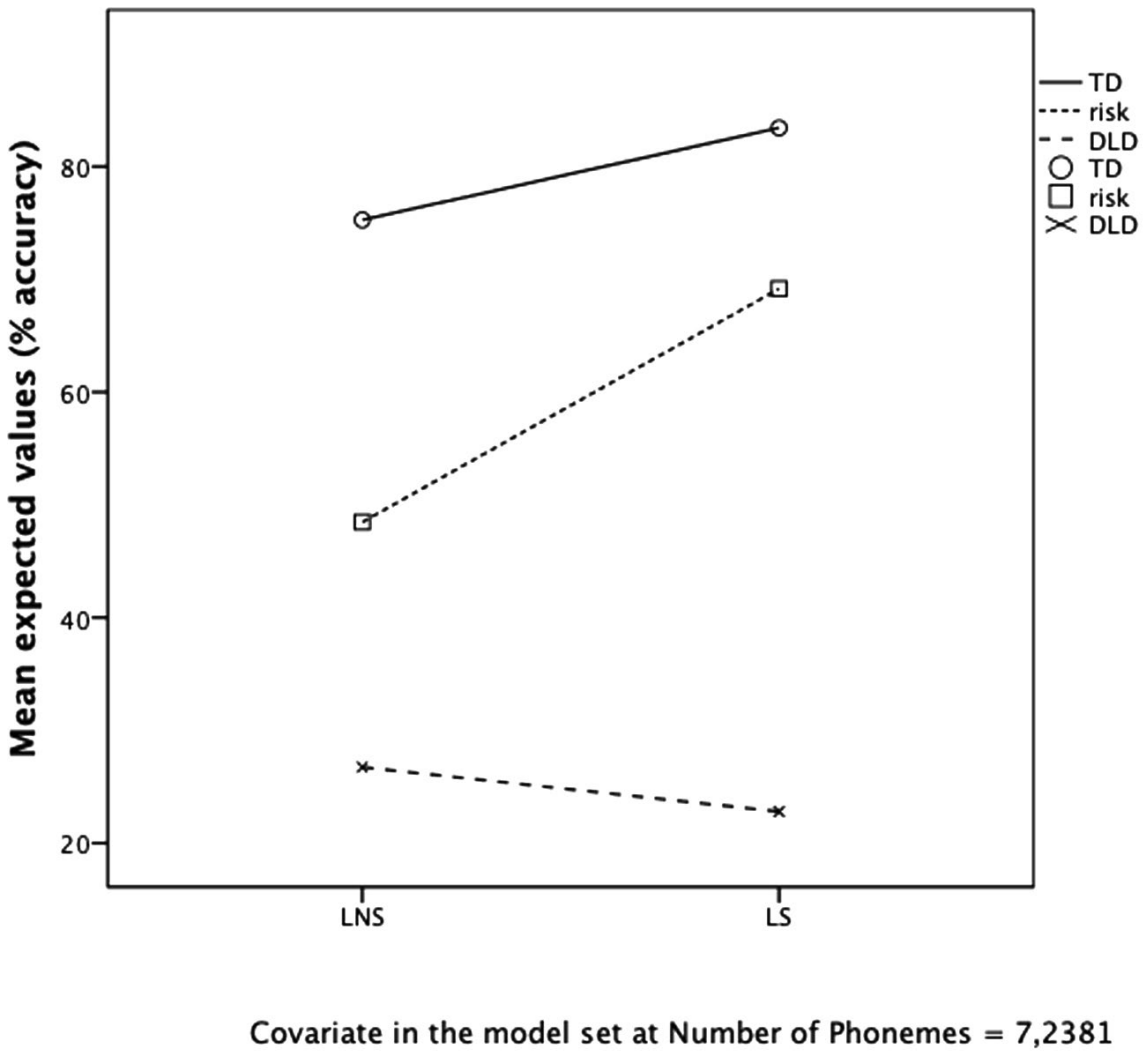
Children’s mean percentage of correctly repeated LNS (language-non-specific, *n* = 9) vs. LS (language-specific, *n* = 11) NWs in the MuLiMi NWRT according to their clinical risk status (TD, at-risk, or DLD).

Since LS and LNS nonwords were matched on number of syllables (*M* = 3.00 for both types) but not on number of phonemes [mean length in phonemes for LS = 7.75, for LNS = 6.56, *t*(19) = −1.662, *p* = 0.113] and this could have affected accuracy, Length in phonemes was set as a covariate in a repeated measures ANOVA with Phonological risk as a within-subject factor (subjects in this case were the NWs) and NW Specificity (LS and LNS) as a between-subject factor. The results showed that the effect of Phonological risk remained significant: *F*(1, 18) = 6.965, *p* = 0.017, *η*^2^ = 0.279 as well as the interaction between Phonological risk and Specificity: *F*(1, 18) = 8.954, *p* = 0.008, *η*^2^ = 0.332. Also the effect of Length almost reached significance, *F*(1, 18) = 4.372, *p* = 0.051, *η*^2^ = 0.195, but its interaction with Phonological risk did not, *p* = 0.327. When repeating the analysis with the three-level variable Clinical status as a within-subject factor [TD, at-risk and DLD, *F*(2, 36) = 14.247, *p* < 0.001, *η*^2^ = 0.442], Specificity as a between-subject factor [interaction Specificity × Clinical status, *F*(2, 36) = 11.333, *p* < 0.001, *η*^2^ = 0.386] and Length as covariate (ns, *p* > 0.1), the results clearly showed (see Figure 3) that LS NWs were highly discriminative of the DLD versus the two other groups (TD and at-risk), whereas LNS NWs discriminated less. Precisely, even if all differences between Clinical status subgroups at post-hoc tests (LSD) were significant for both LS and LNS NWs (*p_s_* ≤ 0.001), the discriminative power (i.e., the differences in subgroup scores) of LS turned out to be significantly higher (covarying for Length) than that of LNS for the comparisons between DLD and the other two groups, *p*_s_ ≤ 0.036, and higher for LNS than LS for the comparison between TD and at-risk children (*p* = 0.025).

A second repeated-measures ANOVA on LS items with Language (German vs. Italian) as within-subjects factor and Phonological risk (with vs. without) as between-subject factor, covarying for age, nonverbal intelligence, and language dominance, yielded a significant main effect of Language [*F*(1, 31) = 8.028, *p* = 0.008] and a significant effect of Phonological risk status [*F*(1, 31) = 51.793, *p* < 0.001, *η*^2^ = 0.626], but no significant interaction effect between Language and Phonological risk status (*p* > 0.05). Among the covariates, only age [*F*(1, 31) = 6.460, *p* = 0.016, *η^2^* = 0.172], but neither nonverbal intelligence, nor language dominance showed a significant effect on repetition performance. Moreover, a highly significant interaction emerged between age and language (*F*(1,32) = 19.385, *p* < 0.001), due to performance on LS-German NWs being rather stable at different ages, but performance on LS-Italian NWs increasing with age.

**Figure 4:**
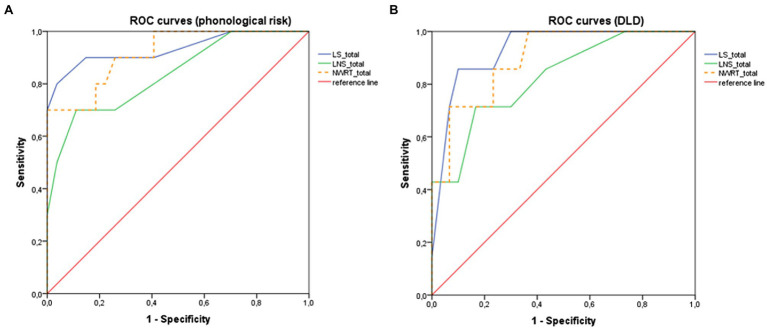
ROC curves for total NWRT, LS, and LNS for the identification of children with **(A)** phonological risk (distinction between phonological risk, *n* = 10, and no risk, n = 27)—AUC = 0.917, 0.933 and 0.837, respectively, and **(B)** DLD (distinction between DLD, *n* = 7, and non-DLD, *n* = 30)—AUC = 0.898, 0.931 and 0.826, respectively.

In order to confirm the discriminative power of the NWRT, an exploratory analysis of diagnostic accuracy (sensitivity and specificity analysis) was performed with respect to the identification of the presence of phonological risk (as opposed to no phonological risk), as well as of DLD (as opposed to TD) in the clinical status classification, in spite of the small sample size. The ROC curves produced by the total NWRT score were very encouraging, with AUC (area under the curve) values as high as 0.917 and 0.898, respectively (*p*_s_ ≤ 0.001). A cut-off of 12.5 would allow for a sensitivity of 0.800 and a specificity of 0.815 in identifying phonological risk, and a sensitivity of 0.857 and a specificity of 0.767 in identifying DLD. When comparing LS and LNS NWs according to their sensitivity and specificity in identifying children with phonological risk and with DLD, respectively, it can be seen that LS produce better ROC curves (AUC = 0.933 and 0.931) than LNS NWs (AUC = 0.837 and 0.826, respectively). Use of LS NWs alone would allow reaching a specificity (with unchanged sensitivity) of 0.900 for DLD identification and 0.963 for phonological risk identification (in the latter case, an alternative cut-off would be associated with a sensitivity of 0.900 and a specificity of 0.852). All ROC curves are presented in Figure 4.

### NW Repetition and Language Test Performance at T1

Performance across all NWRT scales was significantly associated with several standardized measures. Most importantly, the raw scores in the Mottier-test were significantly associated with the percentage of correctly repeated LS NWs (*N* = 37, LS-Italian: *r* = 0.810, *p* < 0.001, LS-German: *r* = 0.624, *p* < 0.001) and the compound scores LS-total (*r* = 0.758, *p* < 0.001) and LNS-total (*r* = 0.621, *p* < 0.001) and with the NWRT total score (*r* = 0.752, *p* < 0.001). These results remained stable when controlling for age and nonverbal intelligence. Children’s scores obtained in the Mottier-test correlated higher (following Meng et al., 1992) with repetition of LS-Italian NWs (*r-difference* = 0.186, *p* = 0.009) than with the repetition of LS-German NWs. The comparison of correlation coefficients for LS total and LNS total with Mottier raw scores did not yield fully significant results (*r-difference* = −0.137, *p* = 0.097). For the two categorical variables verb placement and subject-verb agreement based on the LiSe-DaZ German standardized test, Spearman correlations revealed significant associations with NW repetition performance across subcategories and in the total score (*rho_s_* ranging from 0.348 to 0.494*, p_s_* ≤ 0.029) except for the association between verb placement in the LiSe-DaZ and LNS-total (*rho = 0*.228, *p* > 0.05). All the NWRT scales were also significantly associated with raw scores in the PPVT German standardized vocabulary test (*N* = 37, *r_s_* ranging from 0.397 to 0.600, *p_s_* ≤ 0.015). This pattern was substantially maintained when controlling for age and nonverbal intelligence (*r_s_* > 0.326, *p_s_* ≤ 0.052). Repetition performance across all NWRT scales further correlated with the German CLT compound (noun and verb comprehension) score (*r_s_* ranging 0.354–0.468, *p_s_* < 0.032), whereas the Italian CLT compound score was significantly associated only with repetition performance of LS-Italian (*r* = 0.327, *p* = 0.048) and LNS-total (*r* = 0.395, *p* = 0.016) but not with LS-German (*r* = 0.268, *p* = 0.109) nonwords, and thus not significantly associated with the NWRT total score. This pattern of results remained substantially stable when controlling for age and language dominance, but when adding nonverbal intelligence as a control variable no significant associations were found with the CLT Italian compound score (*r_s_* ≤ 0.209, *p_s_* > 0.220).

Table 4 displays the correlations of both the MuLiMi NWRT and the Mottier-test with the language tests (descriptive values can be found in the Supplementary Table C). While children’s performance in the two LiSe-DaZ subtests, and PPVT raw scores were significantly associated with performance in the Mottier-test as well as in the MuLMi NWRT, only children’s total repetition performance in the MuLiMi NWRT correlated significantly with their receptive lexical skills in Italian and German (CLT performance).

**Table 4:** Correlations between children’s language test performance and the standardized (row 1) and the MuLiMi NWRT (row 2).

	LiSe-DaZ verb placement (from 1 to 4)	LiSe-DaZ subject-verb agreement (from 1 to 4)	PPVT-4 (raw scores)	Italian CLTs (%)	German CLTs (%)
*Performance at T1 (n = 37)*					
Mottier-test (raw score)	*rho* = 0.355, *p* = 0.031	***rho*** ** = 0.482,** ***p*** ** = 0.003**	***r*** ** = 0.412,** ***p*** ** = 0.011**	*r = 0*.255, *p* = 0.128	*r = 0*.316, *p* = 0.057
MuLiMi NWRT total score (%)	*rho* = 0.360, *p* = 0.029	***rho*** ** = 0.494,** ***p*** ** = 0.002**	***r*** ** = 0.519,** ***p*** ** = 0.001**	***r*** ** = 0.369,** ***p*** ** = 0.024**	***r*** ** = 0.434,** ***p*** ** = 0.007**
*Improvement from T1 to T2 (n = 14)*					
Mottier-test (raw score)	***rho*** ** = −0.651,** ***p*** ** = 0.012**	*rho* = −0.491, *p* = 0.074	*rho = −0*.372, *p* = 0.191	*rho* = −0.442, *p* = 0.131	*rho* = 0.052, *p* = 0.860
MuLiMi NWRT total score (%)	*rho* = −0.584, *p* = 0.028	*rho* = −0.360, *p* = 0.206	*rho =* −0.330, *p* = 0.250	***rho*** ** = −0.758,** ***p*** ** = 0.003**	*rho = −0*.286, *p* = 0.321

Bonferroni correction was applied for comparisons of non-mutually correlated variables (alpha set at 0.025). Significant correlations in bold. Parametric correlations are computed for T1 continuous measures considering sufficient sample size, whereas nonparametric correlations (Spearman rho) are computed for ordinal scales (clinical status and LiSe-DaZ scales) and at T2 (*n* = 14) for all measures.

### NW Repetition and Language Experience

For one of the children conflicting information between the parental questionnaire and teachers’ reports emerged regarding his/her amount of Italian output. This participant was thus excluded from the analyses on the Italian output variable. No significant correlations emerged between children’s language dominance scores and their performance on any of the subcategories of NWs, nor did children’s language input (*n* = 36) or output (*n* = 35) in the two languages correlate with any of the NWRT scales (*r_s_* ranging from −0.153 to 0.176, *p_s_* > 0.05).

### NW Repetition and Kindergarten Teachers’ and Parental Questionnaires

Teachers’ global evaluation of children’s language performance correlated significantly with children’s clinical/risk status (*n* = 24, *rho* = 0.803, *p* < 0.001). Particularly teachers’ judgment of children’s productive phonology skills correlated significantly with children’s NWRT scores across all scales (*n* = 23, *rho_s_* ranging from −0.535 to −0.634, *p_s_* ≤ 0.008) except for LS German (*rho* = −0.363, *p* > 0.05).

One of the children had to be excluded from the analysis concerning parental questionnaires since her/his parents never returned the QUIR-DC questionnaire. Children’s clinical/risk status was significantly associated with the responses of their parents in the QUIR-DC general score (QUIR-DC GS, *rho* = −0.393, *p* = 0.018), risk score (QUIR-DC RS, *rho* = 0.361, *p* = 0.031) and family global input score (QUIR-DC FIGS*, rho* = −0.482, *p* = 0.003). Furthermore, children’s performance in all NWRT scales was significantly associated with the scores in the parental questionnaire with the exception of the QUIR-DC FIGS (see Table 5 for an overview). This general pattern was preserved when controlling for age and nonverbal intelligence (CPM *t*-scores).

**Table 5:** Correlations between children’s MuLiMi NW repetition performance at T1 and scores from the parental questionnaire (QUIR-DC, *n* = 36), GS (general score), RS (risk score), and FIGS (family input general score).

	LS-Italian	LS-German	LS-total	LNS-total	NWRT total
QUIR-DC GS	*r* = 0.476, *p* = 0.003	*r* = 0.442, *p* = 0.007	*r* = 0.485, *p* = 0.003	*r* = 0.495, *p* = 0.002	*r* = 0.522, *p* = 0.001
QUIR-DC RS	*r* = −0.400, *p* = 0.016	*r* = −403., *p* = 0.015	*r* = −0.424, *p* = 0.010	*r* = −0.424, *p* = 0.010	*r* = −0.453, *p* = 0.006
QUIR-DC FIGS	*r =* 0.309, *p* > 0.025	*r* = 0.360, *p* > 0.025	*r* = 0.353, *p* > 0.025	*r =* 0.234, *p* > 0.025	*r =* 0.326, *p* > 0.025

Bonferroni correction was applied for comparisons of non-mutually correlated variables (alpha set at 0.025).

### NW Repetition at T1 and at T2

Performance in each subcategory at T1 was significantly correlated with scores obtained within the same subcategory at T2 (see Table 6).

**Table 6:** Correlations between children’s MuLiMi NW repetition performance at T1 and T2 (*n* = 14).

	LS-Italian T2	LS-German T2	LS-total T2	LNS-total T2	NWRT total T2
LS-Italian T1	***rho*** ** = 0.648,** ***p*** ** = 0.012**	*rho* = 0.548, *p* = 0.042	*rho* = 0.637, *p* = 0.014	*rho* = 0.590, *p* = 0.026	*rho* = 0.656, *p* = 0.011
LS-German T1	*rho* = 0.591, *p* = 0.026	***rho*** ** = 0.536,** ***p*** ** = 0.048**	*rho* = 0.615, *p* = 0.019	*rho* = 0.426, *p* > 0.05	*rho* = 0.502, *p* > 0.05
LS-total T1	*rho* = 0.634, *p* = 0.015	*rho* = 0.551, *p* = 0.041	***rho*** ** = 0.644,** ***p*** ** = 0.013**	*rho* = 0.469, *p* > 0.05	*rho* = 0.566, *p* = 0.035
LNS-total T1	*rho* = 0.605, *p* = 0.022	*rho* = 0.376, *p* > 0.05	*rho* = 0.525, *p* > 0.05	***rho*** ** = 0.574,** ***p*** ** = 0.032**	*rho* = 0.545, *p* = 0.044
NWRT total T1	*rho* = 0.602, *p* = 0.023	*rho* = 0.443, *p* > 0.05	*rho* = 0.556, *p* = 0.039	*rho* = 0.562, *p* = 0.037	***rho*** ** = 0.559,** ***p*** ** = 0.038**

Correlations between same-scale scores are highlighted in bold.

**Figure 5:**
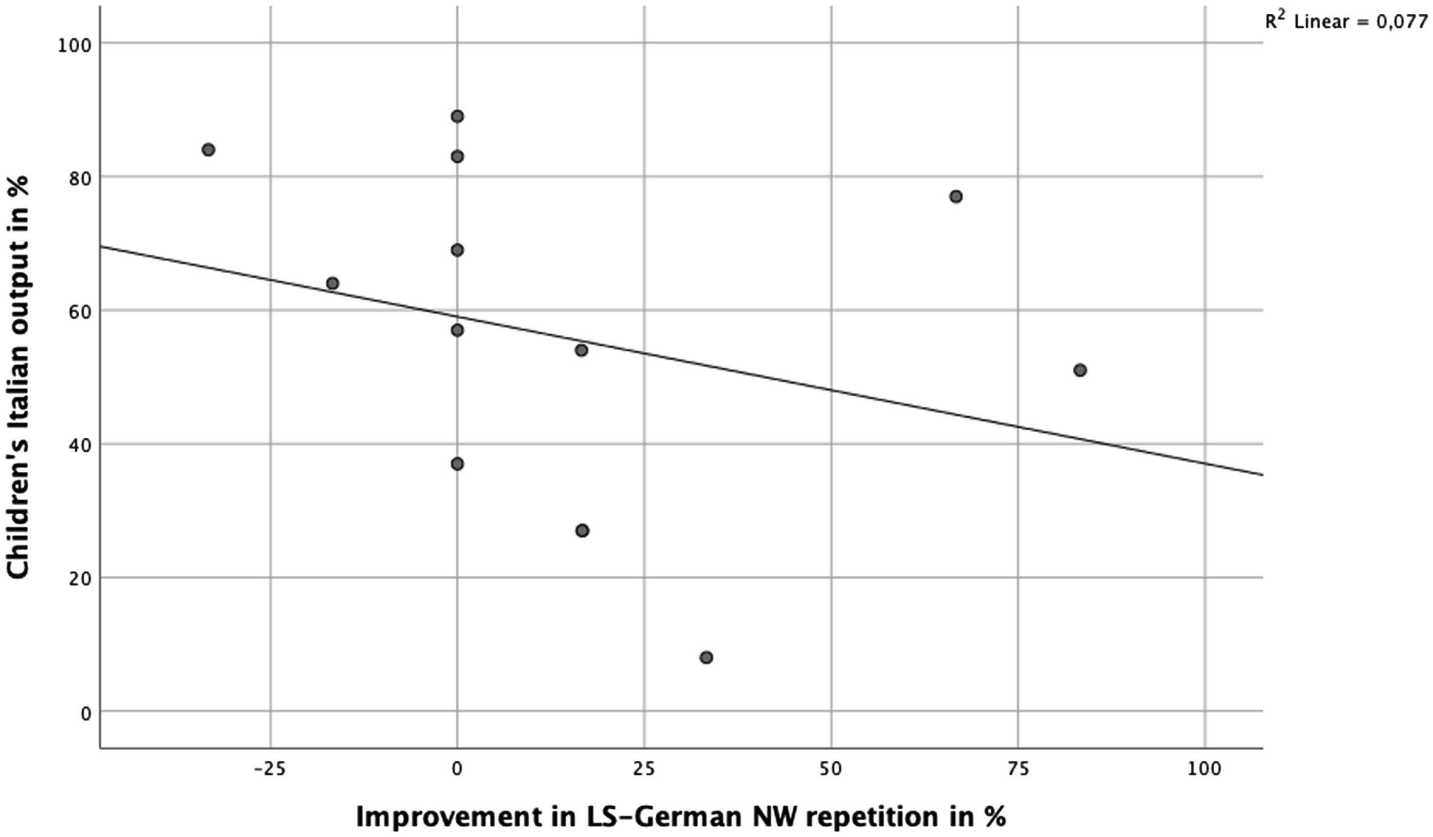
Children’s improvement of correctly repeated LS German NWs from T1 to T2 (*x*-axis) according to their Italian output (*y*-axis). *n* = 13. *Note:* Two data points overlap (Italian output 27%, LS-German NW repetition improvement 16.7%).

Significant associations between performance at T1 and improvement from T1 to T2 were found for the total NWRT score (*n* = 14, *rho* = −0.543, *p* = 0.045) and for LNS-total (*rho* = −0.615, *p* = 0.019). Language dominance was not significantly associated with improvement in NWRT scores from T1 to T2 (*n* = 14, *p_s_* > 0.05). However, an interesting, negative association was found between the amount of Italian output at T1 and the improvement of LS-German scores (*n* = 13, *rho* = −0.553, *p* = 0.050, see Figure 5).

## Discussion

### Answers to the Research Questions

First of all, the discussion will address each of the research questions described in the Introduction.

RQ1: Is MuLiMi NWRT repetition accuracy valid and reliable in the identification of DLD (general risk of DLD or more specifically, risk of a phonological disorder) as assessed by children’s risk status based on German standardized test scores? If yes, is performance in repeating LS items or LNS items more discriminant? And are accuracy scores on the (various types of) NWRT in accordance with parental questionnaires and kindergarten teachers’ subjective ratings?

Reliability of the MuLiMi NWRT was assessed both in terms of inter-rater reliability and of internal consistency. Both indexes were found to be satisfactory. For what concerns validity, children’s performance across all NWRT scales was significantly associated with a wide range of standardized test results. The significant correlations between children’s performance on the MuLiMi nonwords and their scores on further standardized language tests indicate that the task can support the identification of bilingual children’s risk of DLD, irrespective of children’s nonverbal intelligence (CPM *t*-scores). The same results emerge from the correlations between the children’s scores on the NWRT and their position within the classifications expressing clinical status or phonological risk. They further highlight that LS NWs are more discriminant than LNS NWs, especially when the goal is to discriminate between TD and both at-risk and DLD children, or children with phonological risk. ROC curves (as an exploratory analysis considering subgroup size) confirm that the NWRT, and especially LS NWs, may allow the identification of children with either phonological risk or DLD with very good sensitivity and specificity figures.

The validity of the NWRT is also confirmed by the significant correlations found with the QUIR-DC scores and with kindergarten teachers’ evaluations of participants’ language skills. The significant correlations with teachers’ judgment of children’s productive phonological skills show that performance in the NWRT reflects characteristics of children’s real word productions. Our findings actually highlight the particular relevance of using parental and/or teacher questionnaires when assessing bilingual children. Overall then, the present study confirms that NWRTs can help overcome the difficulty of diagnosing DLD in bilingual children, i.e., differentiate between insufficient language exposure vs. actual language impairment.

RQ2: Are there any advantages in using a bilingual NWRT over a monolingual one?

Even though the repetition of LS nonwords in the societal language/L2 or LNS items might already sufficiently give an indication for DLD risk identification in bilingual children, assessment of nonword repetition performance of children’s L1 and L2 provides a more informative picture of the child’s language performance. Actually, the children’s repetition performance with L1-specific nonwords is even more predictive of the children’s clinical or phonological status than their performance with L2-specific nonwords. Moreover, the bilingual NWRT in its full-scale form (21 items) is positively associated with most language tests (of vocabulary and grammar skills, confirming what was found by Hoover and Storkel, 2006; Rispens and Been, 2007; Farabolini et al., 2021). Furthermore, low initial performance on nonword repetition for bilingual children, especially in the case of LNS nonwords, is associated with greater improvement in grammatical and lexical abilities, suggesting temporary, possibly exposure-related delays in phonological abilities. The standardized Mottier-test also correlates with lexical and grammatical measures, but no correlation with the CLTs comprehension subtests is observed and correlations with improvement are limited to German grammatical measures. In a clinically-oriented perspective, it should also be noted that the MuLiMi NWRT has the great advantage of being fully computerized, i.e., it can be used by examiners and therapists with limited or no knowledge of the children’s L1. Although manual correction is required at present, it is envisaged that automatic correction can be implemented in the next future, which will make bilingual assessment usable by virtually any examiner. On the whole, then, the present results highlight the value of NWRTs as predictors of language development and as diagnostic tools especially suited for the bilingual population.

RQ3: Is children’s language dominance correlated with children’s NW repetition accuracy and does language experience (dominance and exposure) influence improvement in NW repetition accuracy?

Similar to what has been found in previous studies (e.g., Dos Santos and Ferré, 2016), TD children achieved higher repetition accuracy than children with DLD for both LS and LNS nonwords. However, differences in informative value for LS vs. LNS NWs were found, suggesting that LS NWs contribute more to the risk identification. More precisely, the discriminative power of LS NWs is particularly high for the identification of DLD children, whereas the discriminative power of LNS NWs appears especially high when the goal is to identify at-risk children among the TD population (even if many of these children seem to overcome their difficulties after a few months). Therefore, both groups of stimuli can be useful for screening purposes. These results confirm the hypothesis that children with DLD are less sensitive to familiarity of incoming speech, both in terms of phonological word structure (Kohnert et al., 2006; Windsor et al., 2010) and in terms of prosody (Archibald and Gathercole, 2007).

Even though dominance differed significantly between the TD and DLD group (TD children more German dominant, DLD children more Italian dominant), no significant association between children’s language dominance and their repetition performance in any of the nonword subcategories was found. Moreover, a measure of German input quality in children’s homes collected through parents’ questionnaires did not correlate significantly with children’s nonword performance in any of the scales (cf. Farabolini et al., 2021). Nonetheless, there was a significant negative correlation between the children’s Italian output and their improvement in the LS German nonwords, suggesting that the more Italian a child speaks on a daily basis, the less (s)he improves in her/his ability to process German phonological stimuli (see Parra et al., 2011). This finding supports the need identified in previous research for adequate language assessment for bilingual children incorporating both languages spoken, especially in early childhood (see Parra et al., 2011; Core et al., 2017) and when children’s lexical knowledge cannot be taken into account (Engel de Abreu, 2011). Notably, this result should not be interpreted as evidence that heritage language use is detrimental for the development of the societal language/L2. Rather, it suggests that both languages need to be taken into account when assessing the child’s language profile, adjusting clinical diagnostic procedures accordingly (e.g., using a bilingual language test like the MuLiMi NWRT). The data show that the MuLiMi NWRT is equally suited for children of varying language experience patterns and language-dominance.

The absence of any influence of language experience on nonword repetition may also suggest that the bilingual children included in this study had already acquired sufficient experience with both of the languages to perform equally well (or badly) on German and Italian language-specific nonwords (cf. Thordardottir and Brandeker, 2013) and thus that the effects of different levels of experience were minimized in this sample.

*RQ4*: Does NW repetition accuracy at T1 predict improvement in overall language performance?

Overall, the significant associations between nonword repetition performance at T1 and T2 across subcategories suggests that the MuLiMi NWRT scores are sufficiently stable across time. Since several months passed between T1 and T2 and since some of the children received Speech and Language Therapy treatment during this period, these correlations cannot be considered as proper measures of test–retest reliability.

Notably, significant associations of children’s nonword repetition accuracy at T1 and their improvement of NWRT scores were found for LNS nonwords but not LS nonword repetition performance. Since performance on LNS at T1 was generally lower than performance on LS nonwords, it is likely that this difference indicates a larger space for improvement for the former than for the latter. Nonetheless, a negative correlation of LNS nonwords at T1 with improvement on the same measure was found. Also, we found greater discriminative power for LS compared to LNS items with respect to phonological risk status. This could indicate that a low performance on LNS items (compared to low performance on LS items) is less likely to reflect a specific impairment of phonological skills. In other terms, there is a greater probability for the repetition of LNS items to improve with time, possibly regardless of clinical intervention.

### Limitations of the Study and Future Directions

The main limitation of the study, due to the difficulty of finding children with DLD belonging to this particular language combination and to limited access to kindergartens and clinical structures during COVID-19-related restrictions, is a relatively small sample size and unequal number of TD children and children with (risk of) DLD. Moreover, the children included in this study do not sufficiently represent the great heterogeneity of language experience scenarios in the bilingual population. Participants were predominantly early sequential or simultaneous bilinguals who most likely had already had sufficient exposure in both languages to perform equally well on LS-German and LS-Italian nonwords. This might be due to the recruitment method with the help of (although not exclusively through) bilingual kindergartens where children receive dual language input. It was therefore not possible to examine how later onset of exposure and acquisition of a second language would influence performance on LS vs. LNS nonwords. The fact that all DLD children were Italian-dominant is also remarkable. This might be a random effect due to small sample size for the DLD group, but it may also suggest that insufficient language exposure to the societal language can be a possible confound in diagnosis, or that it could interact with neurobiological risk factors making the language impairment in the L2 more evident (e.g., in the lexical and/or syntactic domain) and thus enhancing the probability of early diagnosis and treatment. Other possible explanations may call socio-cultural factors into play, the analysis of which is beyond the scope of the present study. These issues could be addressed in future studies where a greater heterogeneity of (bilingual) acquisition patterns is represented in the sample, including simultaneous, early as well as later sequential bilinguals. It would also be interesting to include monolingual participants in order to examine how repetition is affected by the presentation of nonwords whose characteristics go against the phonotactic constraints of their native language.

Furthermore, even if whole word scoring is sufficient to discriminate DLD from TD children, in how far in-depth analyses on syllable- & phoneme-level as well as error analyses could provide additional, useful information will need to be addressed in future studies.

A more general methodological issue and characteristic of most studies on DLD in bilingual population is the circularity between classification and verification. In fact, the classification of children into risk groups is largely based on the results of standard test procedures whose informative value and application with multilingual children is limited. This limitation applies not only to the test procedures with monolingual norms (e.g., the PPVT-4 and Mottier-test) but also to tests with norms for the bilingual population, which (like the LiSe-DaZ) normally refer to a large variety of L1s and to certain bilingual acquisition scenarios only. In this perspective, it would be useful if future studies could incorporate phonological and articulatory tests in both the L1 and the L2.

Finally, the effect of “neutral” intonation in the presentation of LNS nonwords needs to be investigated in more detail since this type of presentation while avoiding language-specific prosodic features on the one hand, on the other hand makes the stimuli sound rather unnatural, a characteristic that might influence repetition performance.

## Conclusion

Summarizing the results of the study with respect to the research questions that were initially raised, it can be concluded that children’s repetition accuracy in the MuLiMi NWRT differs according to their risk status (both clinical/risk status and phonological risk status), confirming the task’s discriminative validity, with a stronger capacity of LS than LNS nonword repetition accuracy to discriminate between children with and without a phonological risk (RQ1). Furthermore, MuLiMi nonword repetition performance is associated with German standardized test scores, confirming the task’s concurrent validity (RQ1). Both teachers’ subjective ratings of children’s language performance and parental reports about children’s language development are largely associated with NW repetition accuracy (RQ1). For bilingual children, the bilingual MuLiMi NWRT is more informative than the monolingual Mottier-test (RQ2). Neither nonword specificity nor children’s language dominance had an impact on NWRT (RQ3), but improvement in NW repetition performance from T1 to T2 did show an effect of language use (RQ4). Preliminarily investigating the MuLiMi NWRT’s predictive validity, children’s repetition performance at T1 correlated significantly with repetition performance at T2 (pointing to stability of the measure) and with improvement (RQ4) which for certain nonword subscales was significantly associated with children’s language use (RQ3).

Thus, in line with previous research (e.g., Pua et al., 2017), a combination of direct (e.g., NWRT) and indirect assessments (such as parental questionnaires and language experience.) seems to be suited for the identification of (a risk of) DLD in bilingual children.

Due to the independence of repetition performance from language dominance, a combination of LS and LNS items provides a satisfactory solution for the identification of language disorders despite the great variety of language experience patterns present in the bilingual population. The present results also suggest that the task is sufficiently robust with respect to the possible impact of nonverbal intelligence and age (within the kindergarten and preschool age range) variations, beyond different language dominance patterns.

For clinical purposes of DLD risk identification and in order to plan and design appropriate intervention strategies, LS nonword repetition performance is the most suitable measure. Repetition of LNS nonwords seems to be more informative with respect to possible improvement in performance, probably due to less specific, more exposure-related impairments. It is thus suggested that a comprehensive assessment should comprise both LS as well as LNS items: the reduction of the items to a relatively small amount of nonwords very carefully selected from the initial list so as to maximize item distinctiveness while preserving validity and reliability allows for relatively fast but highly reliable and informative testing outcomes.

## Data Availability Statement

The raw data supporting the conclusions of this article are available on the Zenodo repository, DOI: 10.5281/zenodo.6519135. Personal data concerning participant children will be made available upon request to the corresponding author. Access will be granted to named individuals in accordance with ethical procedures upon completion of a formal data sharing and collaboration agreement, and approval by the ethics committee and legal authority.

## Ethics Statement

The studies involving human participants were reviewed and approved by Ethics Committee of the Scientific Institute Eugenio Medea, scientific section of the association “La Nostra Famiglia,” via Don Luigi Monza 20, Bosisio Parini (LC) 23842, Italy on 17 June 2019 (no. 43/19); Ethics Committee of the Catholic University Eichstätt-Ingolstadt, Ostenstraße 26, 85072 Eichstätt, Germany, on 21 January 2020 (no. 009-19). Written informed consent to participate in this study was provided by the participants’ legal guardian/next of kin.

## Author Contributions

ME and TB contributed to the conception of the study, definition of the experimental materials for German and Italian, performed data collection, processing and analyses for the study, and contributed to the interpretation of results. ML took care of the conception and definition of the experimental design, assisted in the definition of experimental materials, participated in the interpretation of results, performed statistical analyses, and supervised the whole study. All authors contributed to writing the manuscript, read, and approved the submitted version.

## Funding

This project has received funding from the European Union’s Horizon 2020 program for research and innovation under the Marie Skłodowska Curie Grant Agreement No. 765556 (MultiMind - the multilingual mind) and by the Italian Ministry of Health, Grant RC2021 to ML. The APC was funded by the IRCCS E. Medea, Bosisio Parini, Italy.

## Conflict of Interest

The authors declare that the research was conducted in the absence of any commercial or financial relationships that could be construed as a potential conflict of interest.

## Publisher’s Note

All claims expressed in this article are solely those of the authors and do not necessarily represent those of their affiliated organizations, or those of the publisher, the editors and the reviewers. Any product that may be evaluated in this article, or claim that may be made by its manufacturer, is not guaranteed or endorsed by the publisher.
