# Supplementary material for: A Nonword Repetition Task Discriminates Typically Developing Italian-German Bilingual Children From Bilingual Children With Developmental Language Disorder: The Role of Language-Specific and Language-Non-specific Nonwords
Source: Front Psychol. 2022 Jun 2;13:826540. doi: 10.3389/fpsyg.2022.826540 (PMC9201770; doi:10.3389/fpsyg.2022.826540)
Supplement: Supplementary file 1 [file Data_Sheet_1.pdf]

Supplementary Material

Table A Description of the sample at T1 grouped according to clinical/risk status

	TD (n = 13)	at-risk (n = 17)	DLD (n = 7)
**age** (in months)	*M* = 60.83, *SD* = 10.69	*M* = 58.47, *SD* = 8.46	*M* = 57.86, *SD* = 6.54
**language dominance** (*n* = 36)	*M* = -.246, *SD* = .323<br>German dominant: 53.85%<br>Italian dominant: 7.69%<br>balanced: 38.46%	*M* = -.099, *SD* = .567<br>German dominant: 47.06%<br>Italian dominant: 23.53%<br>balanced: 29.41%	*M* = .201, *SD* = .214<br>German dominant: 0%<br>Italian dominant: 71.43%<br>balanced: 28.57%
**German input** (%)	*M* = 63.71, *SD* = 14.29	*M* = 53.73, *SD* = 22.11	*M* = 42.00, *SD* = 9.90
**German output** (%)	*M* = 71.00, *SD* = 18.24	*M* = 56.00, *SD* = 34.97	*M* = 38.25, *SD* = 14.55
**Italian input** (1 - 4)	*M* = 36.29, *SD* = 14.29	*M* = 46.27, *SD* = 22.11	*M* = 58.00, *SD* = 9.90
**Italian output** (%)	*M* = 29.00, *SD* = 18.24	*M* = 44.00, *SD* = 34.97	*M* = 61.75, *SD* = 14.55
**CPM** (t-scores)	*M* = 56.10, *SD* = 15.45	*M* = 44.90, *SD* = 9.23	*M* = 38.80, *SD* = 6.96
**Mottier** (raw scores, max. 30)	*M* = 15.08, *SD* = 3.62	*M* = 9.47, *SD* = 4.65	*M* = 3.14, *SD* = 3.89
**LiSeDaZ verb placement** (1 - 4)	*M* = 4.00, *SD* = .00	*M* = 3.0, *SD* = 1.55	*M* = 1.75, *SD* = 1.50
**LiSeDaZ subject-verb-agr.**[1] (1 - 4)	*M* = 4.00, *SD* = .00	*M* = 2.73, *SD* = 1.68	*M* = .50, *SD* = .58
**PPVT-4** (raw scores)	*M* = 101.71, *SD* = 20.43	*M* = 85.45, *SD* = 36.36	*M* = 42.00, *SD* = 33.73
**CLT German** (%)	*M* = 91.07, *SD* = 6.61	*M* = 78.41, *SD* = 20.49	*M* = 48.83, *SD* = 23.01
**CLT Italian** (%)	*M* = 63.01, *SD* = 12.04	*M* = 73.16, *SD* = 26.29	*M* = 69.84, *SD* = 20.61
**Teacher global score** (1-21)	*M* = 7.00, *SD* = .00	*M* = 8.91, *SD* = 1.45	*M* = 17.50, *SD* = 3.11
**Teacher prod. phon.**[2] (1-5)	*M* = 1.00, *SD* = .00	*M* = 1.59, *SD* = .66	*M* = 3.25, *SD* = .96
**QUIR GS**	*M* = 107.00, *SD* = 8.06	*M* = 109.18, *SD* = 17.89	*M* = 67.50, *SD* = 22.65
**QUIR RS**	*M* = -30.86, *SD* = 7.71	*M* = -29.36, *SD* = 10.28	*M* = -13.75, *SD* = 10.71
**QUIR FIGS**	*M* = 4.29, *SD* = 1.80	*M* = 4.00, *SD* = 2.24	*M* = -.25, *SD* = .50

[1] LiSeDaz subject-verb-agreement

[2] Teacher productive phonology

Table B Description of the sample at T1 grouped according to their phonological risk status

		no risk (n = 27)	risk (n = 10)
**age**	(in months)	*M* = 59.12, *SD* = 9.72	*M* = 59.20, *SD* = 6.52
**dominance**	(*n* = 36)	*M* = -.108, *SD* = .435<br>German dominant: 44.44%<br>Italian dominant: 18.52%<br>balanced: 37.04%	*M* = -.042, *SD* = .548<br>German dominant: 30.00%<br>Italian dominant: 20.00%<br>balanced: 50.00%
**Italian input**	(%)	*M* = 47.13, *SD* = 17.86	*M* = 41.14, *SD* = 22.34
**Italian output**	(%)	*M* = 44.73, *SD* = 28.41	*M* = 37.57, *SD* = 31.88
**German input**	(%)	*M* = 52.87, *SD* = 17.86	*M* = 58.86, *SD* = 22.34
**German output**	(%)	*M* = 55.27, *SD* = 28.41	*M* = 62.43, *SD* = 31.88
**CPM**	(t-scores)	*M* = 51.53, *SD* = 13.35	*M* = 37.91, *SD* = 5.97
**Mottier**	(raw scores, max. 30)	*M* = 12.81, *SD* = 4.53	*M* = 3.20, *SD* = 2.25
**LiSeDaZ verb placement**	(1-4)	*M* = 3.27, *SD* = 1.39	*M* = 2.71, *SD* = 1.60
**LiSeDaZ subject verb agr.**[1]	(1-4)	*M* = 3.13, *SD* = 1.55	*M* = 1.86, *SD* = 1.77
**PPVT-4**	(raw scores)	*M* = 90.60, *SD* = 32.91	*M* = 65.86, *SD* = 41.37
**CLT German**	(%)	*M* = 81.46, *SD* = 18.52	*M* = 67.63, *SD* = 28.64
**CLT Italian**	(%)	*M* = 79.79, *SD* = 15.56	*M* = 58.05, *SD* = 24.02
**Teacher global score**	(1-21)	*M* = 8.0, *SD* = 1.41	*M* = 13.86, *SD* = 5.15
**Teacher productive phonology**	(1-5)	*M* = 1.23, *SD* = .42	*M* = 2.71, *SD* = 1.11
**QUIR GS**		*M* = 109.13, *SD* = 11.29	*M* = 83.29, *SD* = 30.64
**QUIR RS**		*M* = -30.87, *SD* = 7.75	*M* = -18.71, *SD* = 13.41
**QUIR FIGS**		*M* = 3.80, *SD* = 2.01	*M* = 2.29, *SD* = 3.30

Table C Description of the follow-up sample's (n = 14) improvement scores

**Mottier**	(raw scores, max. 30)	*M* = .77, *SD* = 2.77
**LiSeDaZ verb placement**	(1-4)	*M* = 1.31, *SD* = 1.32
**LiSeDaZ subject verb agr.**[1]	(1-4)	*M* = .08, *SD* = 1.38
**PPVT-4**	(raw scores)	*M* = 22.92, *SD* = 17.16
**CLT German**	(%)	*M* = 13.10, *SD* = 7.26.
**CLT Italian**	(%)	*M* = 4.03, *SD* = 6.13
**LStot NWRT**	(%)	*M* = 8.35, *SD* = 23.83
**LSger NWRT**	(%)	*M* = 11.54, *SD* = 31.46
**LSit NWRT**	(%)	*M* = 5.12, *SD* = 24.28.
**LNS NWRT**	(%)	*M* = 13.69, *SD* = 28.02
**NWRT total score**	(%)	*M* = 10.62, *SD* = 22.68

Table D Overview the list of NWs for the Italian-German MuLiMi NWRT

	**LS German NWs** (*n* = 6)	**LS Italian NWs** (*n* = 6)	**LNS NWs** (*n* = 9)	
			**LNS German NWs** (*n* = 4)	**LNS Italian NWs** (*n* = 5)
**amount of syllables**				
2	[fe’laŋk]	[‘dalmo]	maful	lefum
	[‘nɛːɐ̯lax]			
	[pʰa’molt]			
3	[resol’ant]	[‘spulfarɔ]	nisala	famelep
	[kleŋ’ketɐ̯]	[stal’mo:no]	lifena	fulsamit
				melinak
4	[tulmefo’kans]	[bjɛla’nare]	minalefe	nufalemik
		[maŋke’tale]		
		[rako’denso]		
